# The Reconstructive Toolbox

**DOI:** 10.1055/s-0043-1769619

**Published:** 2023-08-02

**Authors:** Geoffrey G. Hallock

**Affiliations:** 1 Division of Plastic Surgery, Sacred Heart Campus, St. Luke's Hospital, Allentown, Pennsylvania

**Keywords:** reconstructive ladder, reconstructive elevator, reconstructive matrix, reconstructive toolbox

## Abstract

Historically, the approach to any reconstructive challenge, whether intentionally or intuitively, can be seen to follow distinct guidelines that could aptly be called “reconstructive metaphors.” These have been intended to inform us as to the “what, “when” and “where” this attempt can best be achieved. Yet the “how” or means to accomplish this goal, usually also intuitively well understood, in a similar vein can now be expressed to be within our “reconstructive toolbox.” The latter will distinctly mirror our individuality and contain not only the various hardware that we deem essential, but also the means to access whatever technology we may be comfortable with. No toolbox, even if overflowing will ever be full, as potential options and the diversity they represent surely approaches infinity. But the truly excellent reconstructive surgeon will know when their toolbox is in any way lacking, and fears not remedying that deficiency even if the talents of another colleague must be sought, so as always to ensure that the patient will obtain the best appropriate treatment!

## Viewpoint

Just what means “reconstructive” can by itself be a conundrum. Lineweaver has proposed a dichotomy that best defines this term to be both ablative surgery and restorative surgery. 1 Ablative surgery is intended to eliminate if not destroy the etiological agent causing whatever may be the problem. Obviously, many specialties perform such activities; but those who are truly “reconstructive” are distinguished by their capabilities thereafter in providing restoration, whether it be repair of a disrupted wound, rebuilding a defect that requires new parts, or rearrangement of a deformity where there has been a disarray of subunits and their relationships. 1

**Fig. 1:**
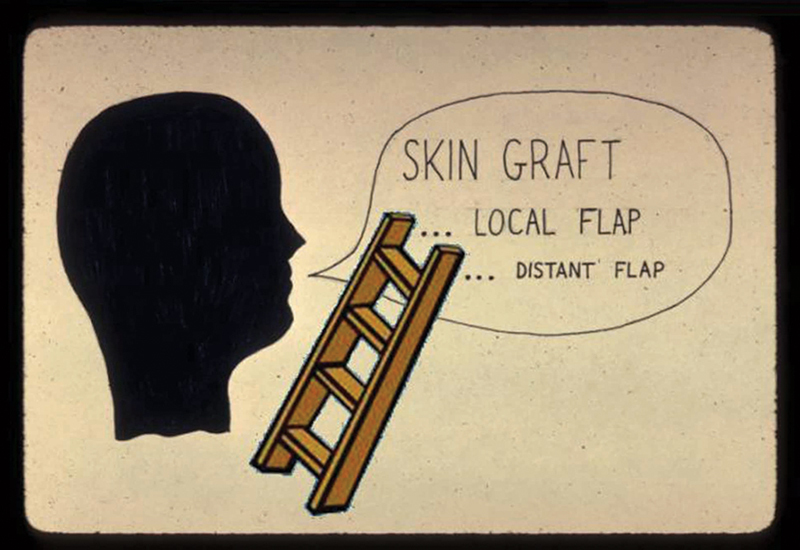
In the beginning, as seen in this resident's vintage slide of the “reconstructive ladder,” the simplest alternative was emphasized, proceeding here downward to more complex options only if absolutely necessary.

**Fig. 2:**
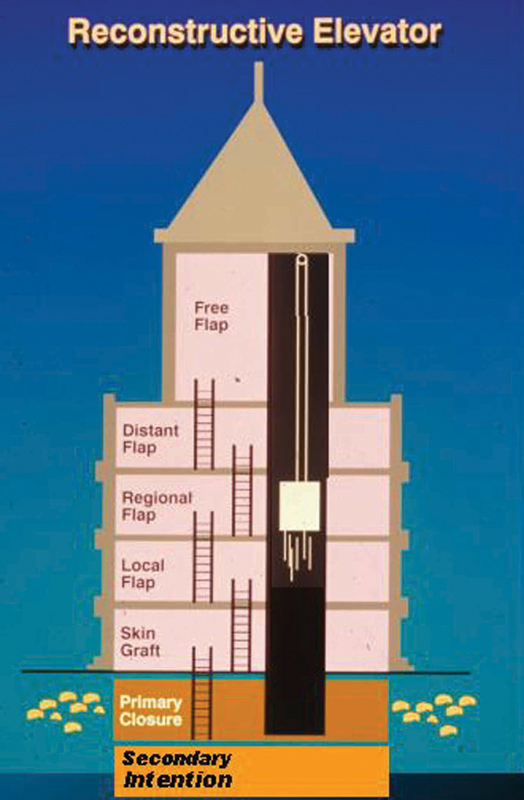
Instead of climbing the ladder from the ground floor to the next, until finally reaching the penthouse, take instead the “reconstructive elevator 2 ” up or down to that floor where the best option can immediately be selected.

Historically, the reconstructive approach to restoration has often relied on the “reconstructive ladder” as a guideline. Gottlieb and Krieger implies this to be an improper extension of the wound closure ladder that probably dates even before the ancient Egyptians. 2 This universal dogma emphasized using the simplest means for obtaining acceptable wound coverage, always bypassing the upward complexity of the rungs of the ladder unless absolutely unavoidable ( Fig. 1 ). But as Mardini et al 3 pointed out in their reconstruction of the reconstructive ladder, the fatal flaw of this principle was a lack of focus on the ultimate functional outcome as well. Probably all “true” reconstructive surgeons so agree and have long recognized that the simplest option may only be a temporary solution, but not always the best for the long term with regard to stability, durability, and reliability while maximizing function. So, the “reconstructive elevator” was established and called for creative rather than just simple sequential thought, allowing the freedom to jump from one rung of the ladder to another in a bidirectional motion to best reach the desired goal ( Fig. 2 ). 2

**Fig. 3:**
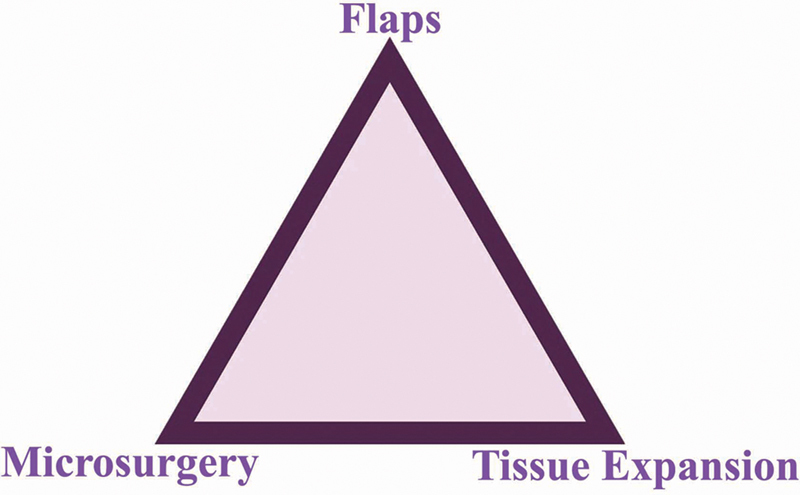
The “reconstructive triangle 4 ” reconstructed the “reconstructive ladder” due to the advent of microsurgery and the concept of tissue expansion, which included both as then the most modern alternatives for a given restoration.

“Change” may be the only commodity that the future can predict, and over time has made the rungs of the “ladder” or floors of the “elevator” in a sense obsolete. Certainly, the advent of microvascular tissue transfers exponentially altered the reconstructive approach. 3 Mathes and Nahai 4 duly recognized this as a facet in their “reconstructive triangle” ( Fig. 3 ). Although this geometric shape in its simplicity overlooked their concurrent opinion that surgical judgement, experience, and technical familiarity were more often needed factors in selecting a reconstructive technique, 4 the complexity of the problem nor the aesthetic and functional requirements for each unique patient were not considered. 5 This latter adage has been reflected in the “reconstructive stages” of Wong and Niranjan, 6 who believed that the difficulty of any restorative challenge depended not on a consideration of the “reconstructive ladder” but instead was directly related to the skill and training of the surgeon as could be witnessed to evolve in their maturation over time.

**Fig. 4:**
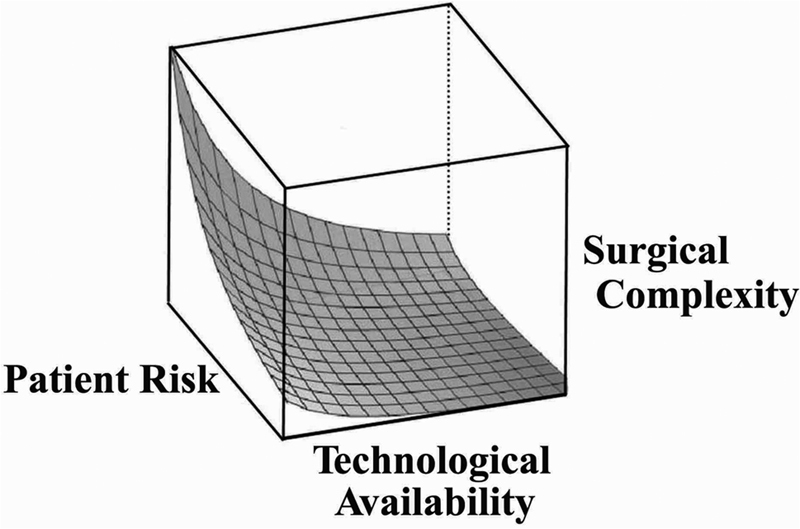
The optimal reconstructive strategy must be individualized using the “reconstructive matrix 5 ” to map a hyperbole unique for each patient. The axes of this three-dimensional model depend on the surgical complexity of the reconstructive challenge, the technological capabilities available for the reconstructive surgeon, and the patient risk factors that would include their comorbidities and expectations for restoration of both form and function.

All the preceding metaphors have each in their own way led to the “reconstructive matrix.” A matrix is a quadratic mathematical form whose axes determine a three-dimensional space incorporating an infinite array of cells each representing a unique possibility. In the reconstructive world, the axes correspond to the perceived surgical complexity of the restorative process, available technological sophistication, and patient-specific surgical risk and expectations. 5 The surgical complexity axis may appear to be directly related to the rungs of the “reconstructive ladder,” but as proselytized in the “reconstructive stages” concept 6 will include an acknowledgment of the skill and experience of the surgeon as well as a recognition of patient variables such as the magnitude of the wound or defect. 5 The exponential explosion of technology more often than not now provides a superior means for accomplishment of the given task. Finally, the benefit for the given patient must always exceed the risk of morbidity in any form, whether at the donor or recipient site. A complete analysis for each patient will project a unique three-dimensional hyperbola within this reconstructive matrix, where each corresponding point on each axis will represent a specific variable ( Fig. 4 ). 5

The latest reconstructive nuance is the “reconstructive grid. 7 ” A framework of rows and columns holds at the bottom a list of the latest and traditional reconstructive choices, as previously many found in the “reconstructive ladder” and “reconstructive elevator.” Above this layer are found rows and columns in a grid delineating the role of judgment, skill, wound complexity, and available resources, as well as observing patient requests and other aspects for their well-being. 7 Perhaps one could say that the boundaries so marked in the “reconstructive grid” in a way are a mirror image of that has already been stated in the “reconstructive matrix.”

**Fig. 5:**
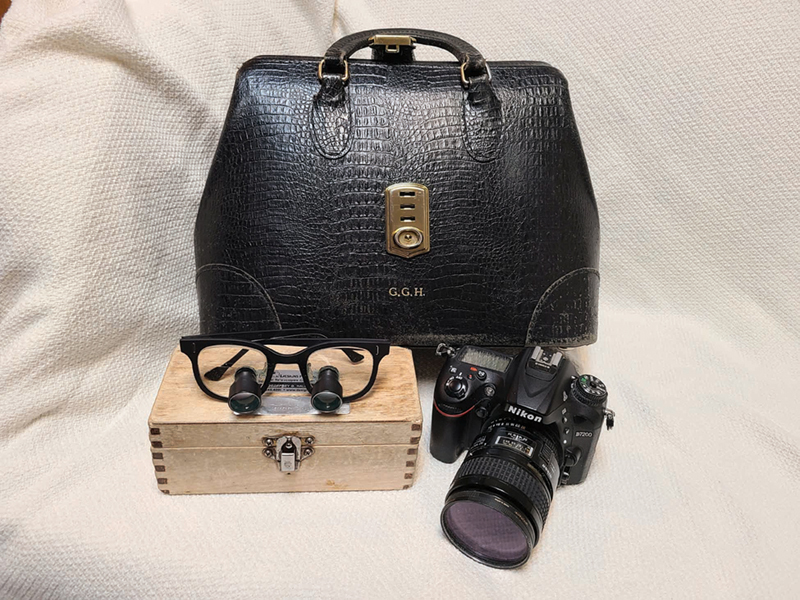
The doctor's handbag historically was not only their medical *toolbox* , but also commanded respect. In simpler times, loupes for vision and a camera for documentation would suffice, if not just a “knife and fork.”

So, it may appear that these aforementioned “reconstructive metaphors” have told us no more than the what and the where should be our choices for any given reconstructive endeavor. Yet they have not told us the how to accomplish this. And should not those means be contained in the “reconstructive toolbox?” Since a toolbox a just a container, then a “reconstructive toolbox” must contain whatever tools are needed for a reconstruction ( Fig. 5 ). 8 9 Once upon a time, this toolbox was filled only by the human brain and the human hand. Some say that still is that most important entity. Yet who knows what other tools like artificial intelligence and technology have already or will lead us to? To maintain relevance, our memory banks must be updated constantly using digital media and online continuing education modules. 10 Basic vascular and lymphatic anatomy and general morphology can be predicted with computed scans, magnetic resonance imaging, indocyanine green, thermography, color Duplex, and now high-frequency ultrasound to allow more security in any preoperative planning. The pursuit of surgery can be held together by swaged on needles, staple guns and staplers, microsutures, anastomotic couplers, and superglue. Loupes, operating microscopes, miniaturized surgical instruments, and next robotic “microsurgery are predicated” on overcoming the limitations of the human hand. 11 Three-dimensional printing or additive manufacturing already provides intrinsic if not virtual models for surgical planning, 12 while also allowing computer-aided design and manufacturing techniques such as to perform more accurate osteotomies. 13 Perhaps with three-dimensional bioprinting or some other form of regenerative medicine, someday flap donor sites will be only those taken off the shelf. 12 Stem cell therapy and gene editing may allow recipient chimerism so that vascularized composite allotransplants will be immunologically practical, 14 but even more pertinent if intrinsic autogenous transformations can be achieved so that “reconstructive” someday may not even be found within the realm of surgery.

## Conclusion

A “reconstructive toolbox” is not just an object that holds the tangible surgical tools necessary to perform what presently we call an operation. Today, the limited capabilities of our most fundamental tools, our brains and hands, have been augmented a thousand-fold with incredible resources and devices. All if placed within this “reconstructive toolbox” now allow us to function more precisely, expeditiously, and more safely, to better meet the restorative and aesthetic expectations that society demands of us. No one alone will be able to clasp all the available tools in their own toolbox, but the best reconstructive surgeon in some way will ensure that the best alternative will not be overlooked. Lest we forget, the best tool in the “reconstructive toolbox” will always be the reconstructive surgeon, and no less.
